# Impact of reading intervention on the phonological awareness of children with autism spectrum disorder: a systematic review and meta-analysis

**DOI:** 10.1590/2317-1782/20242022336en

**Published:** 2024-05-13

**Authors:** Monalysse Francisca Pereira dos Santos, Luana Celly Silva Aprígio, João Victor Silva de Barros Lima, Fernanda Dreux Fernandes Miranda, Cristiano Miranda de Araújo, Karinna Veríssimo Meira Taveira, Cíntia Alves Salgado-Azoni

**Affiliations:** 1 Instituto Santos Dumont – ISD - Macaíba (RN), Brasil.; 2 Universidade Federal do Rio Grande do Norte – UFRN - Natal (RN), Brasil.; 3 Universidade de São Paulo – USP - São Paulo (SP), Brasil.; 4 Universidade Tuiuti do Paraná - Curitiba (PR), Brasil.

**Keywords:** Reading, Autism, Phonological Awareness, Review, Learning

## Abstract

**Purpose:**

To review studies that have intervention in reading with impacts on phonological awareness in children with autism spectrum disorder.

**Research strategies:**

Searches took place until February 2021 in Cochrane, Embase, ERIC (Education Resources Information Center), LILACS (Latin American and Caribbean Health Sciences Literature), PubMed/Medline, Scopus, Web of Science and gray literature databases.

**Selection criteria:**

The review included experimental studies with preschoolers and schoolchildren with ASD. Two independent reviewers selected the studies and, in case of disagreement, a third reviewer was consulted.

**Data analysis:**

Joanna Briggs Institute checklists were used for risk of bias. A random effects meta-analysis was performed and the certainty of the evidence was assessed using the GRADE tool.

**Results:**

Eight studies with some impact on phonological awareness were reviewed. The risk of bias was low and moderate. The certainty of the evidence was low for randomized trials and very low for non-randomised trials. Comparison of pre- and post-therapy on the Preschool Literacy Test (TOPEL) showed that children with ASD improved phonological awareness, with a mean difference between baseline and post-therapy of 6.21 (95% CI = 3.75-8.67; I2 = 0%).

**Conclusion:**

Shared reading and software activities with words and phrases can alter phonological awareness. These results support further research with larger samples and a detailed description of the intervention to observe its effectiveness in phonological awareness.

## INTRODUCTION

Autism Spectrum Disorder (ASD) is included among neurodevelopmental disorders, and for this condition the diagnosis is essentially clinical. According to the Diagnostic and Statistical Manual of Mental Disorders^(1)^, the diagnostic criteria are divided into two groups: (A) deficits in social communication and social interaction, encompassing aspects such as social reciprocity, verbal or non-verbal communication; (B) restricted and repetitive behaviors, interest or activities, such as stereotyped motor movements, echolalia, rigidity to change, fixed interest, hyper- or hyporeactivity to sensory stimuli.

Beyond the language and behavior symptoms, children with ASD may show failures in predictive reading skills, such as phonological awareness^(2-7)^, text interpretation, and difficulty understanding metaphors and implicit information^(8)^. Those that are nonverbal may have difficulty decoding^(9)^, but most studies describe improved performance in reading decoding skills^(10-13)^.

Phonological awareness is a remarkable reading predictive skill that is defined as an ability to identify, manipulate, synthesize and segment the speech sounds^(14)^. Its involves a linguistic knowledge skill that involves the manipulation between graphemes and phonemes at various levels, such as phonemic awareness, rhyme, alliteration^(14,15)^ and it has a relationship with learning to read^(16)^. In addition to this skill, other factors such as oral language, cognition, stimulation environment^(17)^ and other predictors of literacy^(18,19)^ are essential for reading development. Phonological awareness is within the phonological process, just like the lexicon and phonological memory^(20)^.

There is a clear consensus on the effect and influence of the development of phonological awareness in reading skills, especially in decoding. In this way, the bidirectional reinforcement of phonological awareness and reading has been highlighted. Therefore, the better the phonological awareness skills, the greater the reading skills, likewise the better the quality of reading and writing, and there is an increase in phonemic awareness^(14,21-23)^. For above kindergartners level who have already built a strong foundation in early reading skills, tasks such as phoneme segmentation and categorization certainly will be productive (if letters are used)^(24)^.

A home literacy environment that involves a lot of shared reading activities directly helps to improve oral language development, letter knowledge, word decoding, and phonological sensitivity^(25)^. These findings support the relationship between phonological processing skills and literacy development^(21,22)^.

Phonological awareness intervention in children with ASD can be effective in reading development^(3,4,6,26,27)^, considering the diversity of cognitive deficits^(28)^ and the type of strategy in reading to be used, such as shared reading^(29)^.

However, there is still a gap in the understanding of what possible intervention strategies in reading (from decoding to comprehension) can impact on phonological awareness in children with ASD during the school learning period, which can be of relevant scientific contribution to interventional practice in school and clinical rehabilitation^(12)^, which reinforces the need for the present study.

Given the hypothesis that reading intervention can have a positive impact on phonological awareness skills, the guiding question and main objective of this review was to identify and analyze, among the scientific evidence, reading intervention strategies (in different levels) that impact phonological awareness performance in preschool and schoolchildren with ASD.

### Research strategies

This systematic review was developed according to the Preferred Reporting Items for Systematic Reviews and Meta-Analysis^(30)^.

To formulate the objective and guiding question, the PICO (P=population, I=intervention, C= comparison/control, O=outcome) strategy was used, in which the population consisted of preschool and school children with ASD; the intervention focused on reading skills; the comparison involved children with different degrees of ASD and children with typical development; and the outcome was the impact on phonological awareness.

### Selection criteria

The review included experimental (randomized and nonrandomized) studies with preschool and school children with diagnosis of ASD who performed phonological awareness assessment before and after reading intervention, without language and publication time restrictions.

Articles that included assessment of at least one of the phonological awareness skills were considered.

Exclusion criteria included:

1. Studies with adults, elderly, and children out of school age or without a diagnosis of ASD;
2. Studies that do not report reading interventions;
3. Studies that do not assess phonological awareness;
4. Reviews, letters, books, conference summaries, case reports, opinion articles, technical articles, and guidelines.

To apply the search strategies, descriptors with their respective synonyms and free terms were used, combined with Boolean operators, in the following databases: Cochrane, Embase, ERIC (Education Resources Information Center), LILACS (Latin American and Caribbean Health Sciences Literature), PubMed/Medline, Scopus, Web of Science; and in grey literature: Google Scholar, Open Grey, ProQuest Thesis and Dissertation (Appendix A). References were manually searched in all included studies. Searches in electronic databases and grey literature were performed on January 21, 2021, and updated on August 4, 2023. References were managed and duplicate studies were removed using appropriate software (EndNote^®^ X7 Thomson Reuters, Philadelphia, PA).

Two independent reviewers (MFPS and JVL) selected the articles through title and abstract reading. Those selected in this first stage were read in full, applying the eligibility criteria. Differences between reviewers were discussed with the third reviewer (LASC), and the entire team met for the final consensus on eligibility.

Article selection was considered a step-by-step method, and the website Rayyan, from the Qatar Computing Research Institute – QRC^(31)^, was used as a facilitator. Reviewers were blinded in all evaluations.

Two reviewers (MFPS and JVL) independently collected information from the included studies and discussed it with a third revisor (CASA). The articles were organized and presented by author(s); year of publication; type of study / method; sample size; phonological awareness (PA) assessment protocol and skills assessed; intervention program used; outcome and conclusion (Table 1).

**Table 1:** Summary of descriptive characteristics and results of included studies (n = 8)

**AUTHOR(S)**	**TYPE OF STUDY**	**SAMPLE**	**PA ASSESSMENT**	**INTERVENTION**	**OUTCOME**	**CONCLUSION**
Bean et al.^(32)^	Randomized Clinical Trial (secondary analysis)	Autism (n = 22); Developmental language disorder (DLD; n = 23); Typical development (TD; n = 58) = 103	Subtest of the Test of Preschool Early Literacy (TOPEL)^(33)^	Shared reading	Guidance for reading books correlated significantly with gains in knowledge of phonological awareness	Children with ASD showed less orientation towards book reading than their DLD and TD peers. Reading orientation had a critical role in the development of preschool early literacy skills.
Gasamis^(34)^	Randomized Clinical Trial	41 preschoolers	Subtest of the Test of Preschool Early Literacy (TOPEL)^(33)^	Interactive book reading	Gains in phonological awareness	Changes took place in PA from pre- to post-test, but children who had greater PA in the pre-test had less gain in the post-test because they had already developed the skills under study.
Heimann et al.^(35)^	Nonrandomized Clinical Trial	30 children (11 with ASD, 9 with mixed disabilities, 10 neurotypic)	Swedish instrument^(36)^	Alpha Program	Gains in phonological awareness in the training period	The gain was not consolidated for children with ASD as there was a decline after the training period.
Hudson et al.^(37)^	Randomized Clinical Trial	47 (IBR), 42 (PA), and 44 (BAU) = 133	Subtest of the Test of Preschool Early Literacy (TOPEL)^(33)^	Interactive book reading	Interactive book reading showed no response in PA	Only the intervention that focused on PA showed effect on this parameter.
Kimhi et al.^(38)^	Nonrandomized Clinical Trial	5 participants (4 boys and 1 girl)	Naming of Hebrew letters and letter sounds to assess the understanding of grapheme-phoneme relationships, supported by an alphabet ruler showing consecutive letters	Shared reading	After the intervention, 3 children acquired grapheme-phoneme knowledge and knowledge of the complete alphabet	Significant effect on phonological awareness.
Nally^(39)^	Randomized Clinical Trial (control group)	The final sample included 26 participants, divided between control and experimental groups by ASD severity	Dynamic Indicators of Basic Early Literacy Skills (DIBELS Next)^(40,41)^	Interactive reading - virtual program	Gains in phonological awareness and target sound	With the Headsprout® program, participants showed greater overall gains, more specifically in phonological awareness and target sound.
Pamparo^(42)^	Nonrandomized Clinical Trial	14 students	Subtest of the Test of Preschool Early Literacy (TOPEL)^(33)^	Dialogic reading	Students’ knowledge about phonological awareness did not differ between pre-test, post-test, and follow-up	Dialogic reading may have not been effective in improving students’ phonological awareness skills due to the lack of interactions focusing on phonology.
Tjus et al.^(43)^	Nonrandomized Clinical Trial	30 children with ASD	Swedish instrument^(36)^	Multimedia program for sentence construction with 10 lessons	Highly experienced gains were observed for both reading and phonological during the treatment phase.	The intervention had side effects for the gain in reading level, response speed with conscious and phonological sentences.

Capture: PA = Phonological Awareness; ASD = Autism Spectrum Disorder

Risk of bias assessment: The risk of bias of the included studies was assessed using the critical appraisal checklist from the Joanna Briggs Institute for randomized^(44)^ and nonrandomized studies^(45)^. Two reviewers (MFPS and JVL) assessed the risk of bias separately and judged the included articles, marking each assessment criterion with “yes”, “no”, “uncertain”, or “not applicable”. Risk of bias was rated as high when the study reached 49% “Yes” answers; moderate when the study reached 50% to 69% “Yes” answers; and low when the study reached more than 70% “Yes” answers. When necessary, disagreements were discussed with the third (LASC) and fourth reviewer (FDMF). RevMan 5.4 software (Review Manager 5.4; The Cochrane Collaboration) was used to generate the figures.

### Data analysis

Data referring to the mean (M) and standard deviation (SD) of the included studies were collected. Then, the effect measure was calculated as the difference between means (MD) with pre-, post-intervention, and follow-up comparative data, considering the normative and non-normative data of the tests used in the studies.

A random effects meta-analysis method was performed, with studies weighted by the inverse variance method, to estimate the difference between means from pre- to post-therapy. Heterogeneity was calculated by the inconsistency index (I^2^), and variance by Tau^2^, estimated by the DerSimonian-Laird method. Additionally, 95% confidence intervals were generated (95% CI) and the significance level was set at 5%. The meta-analysis and forest plots were performed using Review Manager software version 5.4.1. (Cochrane Community, London, UK).

The certainty of evidence was analyzed using GRADE® (Grading of Recommendations Assessment, Development, and Evaluation)^(46)^ which is a quality scoring system^(47)^. Two reviewers (MFPS and KVMT) judged the following aspects: risk of bias, inconsistency, indirect evidence, imprecision, and publication bias for the different results. The level of evidence was classified as high, moderate, low, or very low. Disagreements were resolved by consensus, and a third reviewer (CASA) was consulted when necessary.

## RESULTS

**Figure 1:**
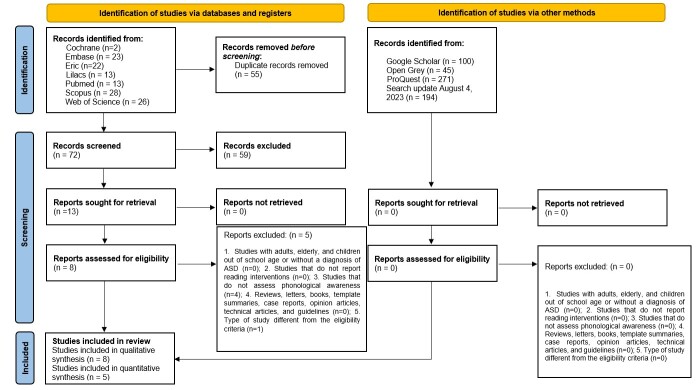
PRISMA 2020 flow diagram for new systematic reviews which included searches of databases, registers and other sources

A total of 543 references were retrieved by the search strategy in the ten electronic databases, of which 462 remained after the removal of duplicate references. After reading the titles and abstracts (phase 1), 13 articles were selected for full reading (phase 2), of which 5 were excluded. Thus, 8 articles were included for qualitative and 5 to quantitative synthesis (Figure 1 and Appendix B). No additional articles from the reference list and grey literature were included.

On August 4, 2023, a new search was carried out to update new articles. A total of 194 were found, therefore, all were excluded after reading titles and abstracts.

Of the studies included, one was published in 1995^(35)^, one in 1998^(43)^, one in 2012^(42)^, two in 2017^(37,38)^, two in 2018^(34,39)^ and another one in 2020^(32)^.

Sample sizes across studies ranged from 5 to 133 participants, most of whom were male. Regarding the types of study, only experimental studies were selected, four randomized^(32,34,37,39)^ and four nonrandomized^(35,37,38,42,43)^.

Among these studies, four^(32,34,37,42)^ used the Phonological Awareness Subtest of the Test of Preschool Early Literacy - TOPEL and two^(35,43)^ used the *Fonologisk medvetenhet. Handledning för kartläggning och utveckling*. Six studies used shared reading stimulation as intervention, which consists of an adult reading aloud to one or more children^(32,34,37-39,42)^ and two used a software (Alpha) to stimulate individual words and phrases production^(35,43)^.

Four studies^(32,34,38,39,43)^ demonstrated some impact on phonological awareness after the reading intervention, unlike two others^(37,42)^ that showed no improvement after the intervention and one^(35)^ showed decline in phonological awareness after the end of the training period. Table 1 provides information regarding the characteristics of the included studies.

**Figure 2:**
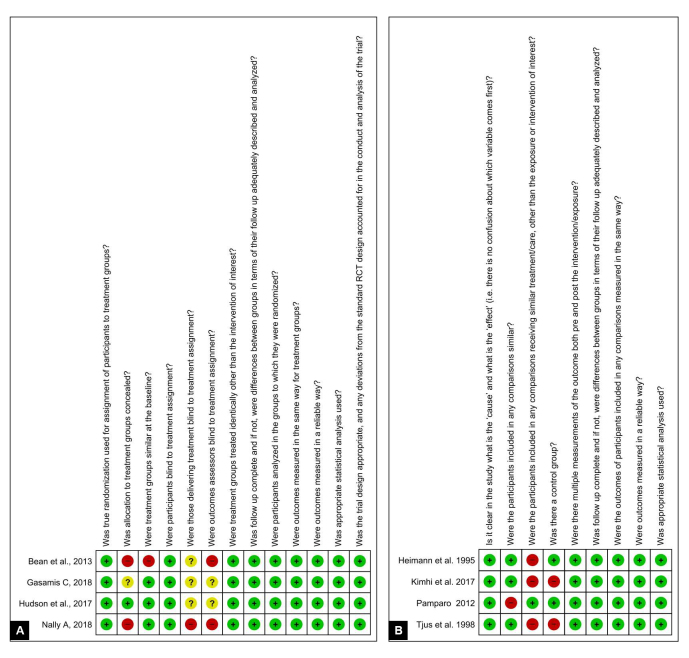
Analysis of randomized (A) and nonrandomized (B) studies

With regard to the risk of bias in individual studies, one study had moderate risk^(32)^ and seven studies had low risk^(34,35,37-39,42,43)^ (Figures 2A and 2B; and Appendices C1 and C2).

As for the methodological quality of the studies, analyzed by the appraisal checklist from the Joanna Briggs Institute (JBI), among the four nonrandomized studies, the study by Kimhi et al.^(38)^ and Tjus et al.^(43)^ had seven “yes” answers, while the study by Pamparo^(42)^ and Heimann et al.^(35)^ had eight “yes” answers. In the evaluation of randomized studies, one study had 9 “yes” answers^(32)^, two studies^(34,39)^ had 10 “yes” answers, and the study by Hudson et al.^(37)^ had 11 “yes” answers. Appendix C summarize the findings regarding the JBI tool.

For the randomized studies included in this review, methodological limitations consisted of the lack of blinding for group allocation^(32,34,37,39)^, for intervention facilitators, and for outcome evaluators^(32,34,37,39)^, as well as the lack of similarity between treatment groups^(32)^, and the reduced number of participants. For nonrandomized studies, limitations consisted of the lack of comparison with a similar group^(31)^ or other than exposure or intervention of interest^(35,38,43)^, any comparisons beyond the intervention, as well as the absence of a control group^(38,43)^. Figure 2 summarizes the evaluations obtained through the JBI tool.

Among the nonrandomized studies, Heimann et al.^(35)^ provided important information about a computer-aided instruction intervention. The authors aimed to investigate the effects of these instructions from a highly motivating and interactive multimedia environment (“Alpha” program) when teaching reading and writing for children with ASD. The intervention was performed according to four main activity models: individual words (IW), creating sentences (CS), testing words (TW), and testing sentences (TS), and children would advance from one activity to the other as each domain was reached. Children’s phonological awareness was assessed using a Swedish instrument before and after the intervention. All children increased their means during the training period (Start-Post 1) which is evident by the combined result for Group A and Group MH displayed. Significant gains are observed both during the training period proper, t(13) = 2.7, p < .02, and from Start until Post 2 at the follow-up evaluation, t(13) = 1.99, p < .05. If each group is analyzed separately, we note significant gains for both Groups A and NP from Start to Post 1, t(7) = 2.5 and t(8) = 2.48, respectively, p < .03), and for Group MH from Start to Post 2, t(5) = 2.29, p < .05.

The study by Tjus et al.^(43)^ the phonological awareness (sound synthesis and segmentation) was also assessed using a Swedish instrument. There was no comparison between groups, only the group of children with ASD at three times (baseline, training and follow-up). A comparison between the baseline period (mean -0.8, SD 7.3) and the intervention period (mean 4.6, SD 6.1) yielded a significant contrast (t (12) = 2.26, p<0.03), as did the comparison between the follow-up period (mean 5.1, SD 6.4) and the baseline (mean -0.8, SD 7.3) (t (12) = 1.79, p<0.05) These effects in reading and phonological awareness are also evident by the results expressed in raw scores.

Kimhi et al.^(38)^ investigated a national natural literacy program aimed at promoting literacy predictive skills, including phonological awareness-letter sounding. The authors assessed phonological awareness just through the grapheme-phoneme relations, using a ruler that displayed the 22 letters of the Hebrew alphabet without the final five letters. Children were asked to sound each phoneme (score of 0-22). Among the five kindergarten children with ASD, three had no grapheme-phoneme knowledge (0/22), one had basic knowledge (8/22), and another had advanced knowledge (18/22). One of the children who did not have knowledge and another who had basic knowledge progressed after the intervention, reaching the total score (22/22), as well as the one who already had advanced knowledge (22/22). Grapheme-phoneme knowledge differed significantly between the pre-test (M = 5.20, SD = 7.94) and the post-test (M = 16.2, SD = 14.78). The nonparametric Wilcoxon signed rank test revealed significant differences from the pre-test/post-test group for grapheme-phoneme knowledge (Z = 1.60, P < 0.05).

The other nonrandomized study, Pamparo^(42)^, examined the effect of dialogic reading strategies on early literacy outcomes in 14 preschoolers with ASD. The results of the dialogic reading study consist of socially valid and effective strategies to improve the language of children with ASD, but the knowledge of phonological awareness did not differ from pre- to post-test in this group.

Among the randomized studies, the shared reading intervention strategy of Bean et al.^(32)^ compared three groups, one with autism spectrum disorder (ASD), the second with developmental language disorder (DLD), and the third with typically developing children. The groups were randomly distributed, and the mean age of children was 4 years. When correlating the findings of the groups with the skills studied and the book-reading orientation, the ASD group showed less significance in phonological awareness than the DLD group. However, the results indicated that book-reading orientation correlated significantly with the residual gains used by children in phonological awareness. Furthermore, book-reading orientation was a unique predictor of emergent literacy skills such as phonological awareness, even when controlling for ASD, DLD, and oral language.

Gasamis^(34)^ observed how tutors of a literacy program in the school environment encouraged shared reading in 41 children with ASD. Among the assessments that took place before and after the intervention, phonological awareness improved from pre-test (M = 71.10; SD = 22.80) to post-test (M = 81.73; SD = 25.88), with a gain corresponding to (M = 10.15; SD = 13.81). However, children who performed better in phonological awareness in the pre-test had less gain in the post-test.

Hudson et al.^(37)^ compared two types of intervention, one directed towards interactive book reading (IBR) and the other towards phonological awareness (PA). The study included a series of three consecutive randomized controlled trials, with a control group (BAU). The authors compared the performance in language skills between the IBR and control groups; PA and control groups; and IBR and PA groups. Descriptive measures showed improvement in phonological awareness in the IBR (pre-test: M = 75.21; SD = 15.33; post-test: M = 83.23; SD = 15.83; gain = 8.02), PA (pre-test: M = 79.21; SD = 15.22; post-test: M = 91.74; SD = 22.65; gain = 12.53), and BAU groups (pre-test: M = 77.88; SD = 18.94; post-test: M = 82.11; SD = 17.96; gain = 4.23), but phonological awareness was more effective for children who were stimulated in the PA intervention.

In the randomization performed in the study by Nally^(39)^, the authors conducted a reading intervention for children with ASD. The procedure relied on instructions to parents for monitoring and support at home, using the computer program Headsprout^®^, according to the Applied Behavior Analysis approach. The intervention was carried out for 10 weeks for the experimental group. In the post-test evaluation, these children showed gains in pseudoword reading skills, phonemic segmentation fluency, first sound fluency, first sound level, and first word level. On the other hand, this group presented worse reading and phonemic segmentation fluency (PSF) in relation to the control group.

Six studies were included in the meta-analysis; however, the study by Hudson et al.^(37)^ presented three groups eligible to be included in the analysis. All assessments were performed using the Test of Preschool Early Literacy (TOPEL). Two studies^(35,43)^ were excluded in the meta-analysis because different sub-items in the instrument were used to compare phonological awareness.

**Figure 3:**
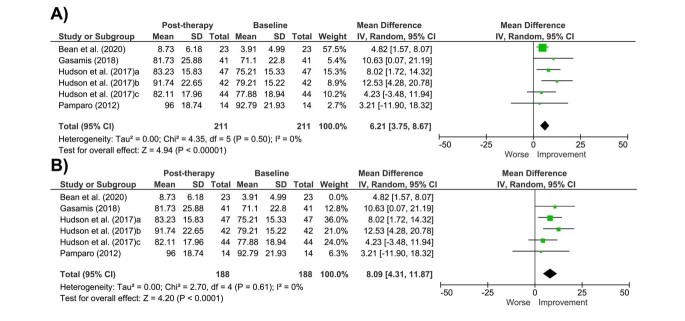
Forest plot graph to assess the difference between pre- and post-therapy means of phonological awareness of individuals with ASD: (A) Analysis with all eligible studies; (B) Sensitivity analysis

The comparison of pre- and post-therapy data regarding the TOPEL showed that children with ASD improved in phonological awareness, with a mean difference of 6.21 (95% CI = 3.75-8.67; I^2^ = 0%) from baseline to post-therapy (Figure 3).

Although the data did not show heterogeneity (Tau^2^ = 0.00; p = 0.50), the estimate reported by Bean et al.^(32)^ showed lower variance, resulting in greater weight in the analysis. Hence, a sensitivity analysis was performed to ensure data robustness. Even after the sensitivity analysis, the results point to improvement in phonological awareness in children with ASD (MD = 8.09; 95% CI 4.31-11.87; I^2^ = 0%) (Figure 3).

Only two exclusion criteria took place: studies that did not assess phonological awareness^(48-51)^, and study design different from the eligibility criteria^(52)^. One study showed potential for review, but it was excluded for not specifically assessing phonological awareness^(51)^.

The study by Colcord et al.^(52)^ aimed to observe the effect of the reading intervention, through peer-assisted learning strategy (PALS) instruction, in which two 8-year-old children with ASD worked together with support from an interventionist to assist in the learning process for 5 weeks. The activities involved stimulation of word recognition, accurate reading fluency and general word fluency in addition to reading comprehension.

Uccheddu et al.^(51)^ presented a randomized experimental design, with a sample of 9 children with ASD between 6 and 11 years old, divided into control and experimental groups. The research described a training in which participants had to read for dogs. However, the authors used a comprehensive protocol for the assessment of phonological awareness, without specific data on its change from pre- to post-intervention. Thus, the article was excluded from this review.

The overall certainty of evidence identified using the GRADE tool^(47)^ was low for randomized clinical trials and very low for nonrandomized clinical trials due to the following reasons: “severe” high risk of bias, as none of the studies performed sample randomization; lack of allocation; treatment evaluators were not blinded; inconsistency due to the use of a different assessment protocol; inaccuracy related to the small sample and the sizes and number of events. Publication bias was undetected, as there was an effort to search the entire literature on the subject, including grey literature. The potential conflict of interest of the included studies was also undetected. Two studies^(35,43)^ were not entered into the certainty of evidence because they were not included in the meta-analysis (Table 2).

**Table 2:** Analysis of information quality through GRADE Research question: Which reading intervention strategies that impact the performance of phonological awareness performed in preschool and school children with ASD?

**Certainty assessment**						
**Studies**	**Risk of bias**	**Inconsistency**	**Indirectness**	**Imprecision**	**Publication bias**	**Overall certainty of evidence**
4 RCTs	serious a	not serious	not serious	serious b	none	⨁⨁◯◯ LOW
4 NRCTs	serious c	serious d	not serious	serious ^b^	none	⨁◯◯◯ VERY LOW

a Problems with randomization and allocation concealment;

b The sample size or the number of events does not meet the optimal information (Cochrane handbook, Chapter 14);

c The evaluators of the treatment results were not blinded;

d Different questionnaires were used for the evaluation

This systematic review with meta-analysis investigated the impact of reading intervention on the phonological awareness of children with ASD. The included studies did not show heterogeneity, and the result showed significance even after sensitivity analysis. Phonological awareness improved after reading intervention, demonstrating intervention effectiveness for this response. Most of the studies used the same intervention, i.e., shared reading strategy, facilitated by an adult to help children’s language development, especially with children with ASD^(29,53,54)^.

Studies with shared reading in children with ASD have been described as a way to socially engage them, to improve oral language and story comprehension^(17,54-56)^, but in this review it was possible to verify its effectiveness in improving phonological awareness.

The positive impact on the development of phonological awareness from this intervention can lead to success in the development of the child’s literacy, as this skill is an important predictor for learning to read and write^(14-16,57)^. Although this latter study did not direct the intervention towards phonological awareness itself, it is noteworthy that directly stimulating this skill can also be efficient in the development of reading, especially in the word recognition, in this clinical group^(3,21)^.

Some studies intended for this review did not provide detailed information about the format of assessment of participants, how the interventions occurred, nor which specific phonological awareness skills improved or changed in any way. This fact may correlate with the risk of bias of studies being assessed by the JBI tool as moderate^(32)^ and low^(34,35,37-39,42,43)^. Furthermore, according to GRADE^(47)^, methodological quality was low for randomized clinical trials and very low for nonrandomized clinical trials. This reinforces that the lack of detail regarding intervention in individuals with ASD does not have to do only with the choice of the method itself, but also with many other conditions.

In contrast, one of the studies explained in detail how the intervention occurred and identified aspects that improved in the test parameters used (phoneme segmentation, synthesis and deletion). The activities of the intervention program used are carried out to stimulate the development of basic words reading and writing, vocabulary and sentence construction. All started with the test of individual words (nouns), and after reaching the score for this test, they proceeded to the construction of simple sentences applying the learned vocabulary, and joining the verbs, where animations representing the elaborated phrase appear. Therefore, after mastering this step, it proceeded to the phrase test^(35)^.

As the performance of predictive skills presents an interdependent relationship, it is important that professionals working in the context of individuals with ASD, including speech therapists, teachers, psychologists, and educators, are aware of and attentive not only to phonological awareness, but also to another reading-related skill, which is phonological memory because it works together with phonological awareness in learning to read^(2,29,58)^.

Some methodological limitations of this review should be considered. Despite the use of shared reading as an intervention, different approaches and strategies were used in groups of children with ASD. Regarding the risk of bias of the included studies, there is no information on blinding and allocation of intervention groups in most studies.

Overall, reading interventions in children with ASD are still being poorly investigated when it comes to responses regarding specific skills such as phonological awareness. There is more research on the oral language of this audience, and limited evidence on learning difficulties. Many studies were excluded after the complete article reading stage, as they did not contain the established eligibility criteria. Furthermore, educators have expanded the search for ways that better direct the effectiveness of the learning process in children with ASD, although the possibility of acting on reading and writing development also involves the work of the speech therapist.

In the development of intervention strategies aimed at children with ASD, it is paramount to analyze not only chronological age, but also mental age, cognitive development, and other developmental indicators, since these factors may influence the performance on responses. The studies included in this review described participants’ chronological age, but only two mentioned mental age^(32,39)^ two used other parameters to observe participants’ development and cooperation in a test on vocabulary, sentence production, and understanding of simple orders^(34,37)^ and the other two considered only the vocabulary^(38,42)^.

Extrapolation of results is not possible due to specific characteristics of the cognition of children with ASD, which is a heterogeneous condition. In this study, children who performed the interventions were in regular schools, which can serve as further evidence that it is possible to implement reading intervention programs with positive data on phonological awareness in children with ASD.

Even without a consensus among studies regarding the positive response for phonological awareness, shared reading proved to be an intervention that changes phonological awareness skills.

Despite the similarity in the type of study and evaluation criteria and the possibility of conducting a quantitative approach with meta-analysis with more accurate data, research with larger samples is needed. Further studies should address a better description of the intervention, including frequency and duration, with good methodological quality so as to improve the analysis of effectiveness, as these difficulties are already expected for this audience. Thus, the scope of studies will enable the clinical application of evidence-based practice.

## CONCLUSION

This review also contributed to increasing the visibility of the importance of interventions to promote reading in children with ASD from the investigation of the strategies used and their adequacy to serve this audience. Studies like this enable more effective help for people inserted in the daily context of these children, such as health and education professionals, with positive consequences for comprehensive care. We suggest that future studies verify the effects of phonological awareness after interventions at different reading levels, as well as the stimulation of this skill to help develop decoding skills in children with ASD, an important step in reading processing.

## FURTHER INFORMATION

The protocol was registered on the PROSPERO website (International Prospective Register of Systematic Reviews, Center for Reviews and Dissemination, University of York) under Number CRD42021238697.

## Appendix A Search strategies used in databases and literature

**Table:**

**Database**	**Search Strategies**
Cochrane	((“Autism Spectrum Disorder” OR “Autism Spectrum Disorders” OR “Autistic Disorder” OR “Kanner's Syndrome” OR “Kanner Syndrome” OR “Kanners Syndrome” OR “Infantile Autism” OR “Autism” OR “Early Infantile Autism” OR “ASD”) AND (“reading”) AND (“phonological awareness” OR “phonological awareness skills”))
EMBASE	('autism spectrum disorder'/exp OR 'autism spectrum disorder' OR 'autism spectrum disorders'/exp OR 'autism spectrum disorders' OR 'autistic disorder'/exp OR 'autistic disorder' OR 'kanner syndrome'/exp OR 'kanner syndrome' OR 'kanners syndrome' OR 'infantile autism'/exp OR 'infantile autism' OR 'autism'/exp OR 'autism' OR 'early infantile autism'/exp OR 'early infantile autism' OR 'asd'/exp OR 'asd') AND ('reading') AND ('phonological awareness'/exp OR 'phonological awareness' OR 'phonological awareness skills')
ERIC	((“Autism Spectrum Disorder” OR “Autism Spectrum Disorders” OR “Autistic Disorder” OR “Kanner's Syndrome” OR “Kanner Syndrome” OR “Kanners Syndrome” OR “Infantile Autism” OR “Autism” OR “Early Infantile Autism” OR “ASD”) AND (“reading”) AND (“phonological awareness” OR “phonological awareness skills”))
Google Scholar	“Autism Spectrum Disorder” OR “Autistic Disorder” AND “reading” AND “phonological awareness” filetype:PDF
LILACS	((“Trastorno del Espectro Autista” OR “Trouble du spectre autistique” OR “Transtorno do Espectro Autista” OR “Transtorno de Espectro Autista” OR “Transtorno do Espectro do Autismo” OR “Autism Spectrum Disorder” OR “Autism Spectrum Disorders” OR “Autistic Disorder” OR “Kanner's Syndrome” OR “Kanner Syndrome” OR “Kanners Syndrome” OR “Infantile Autism” OR “Autism” OR “Early Infantile Autism” OR “ASD” OR “Transtorno Autístico” OR “Autismo” OR “Autismo Infantil” OR “Síndrome de Kanner” OR “Trastorno Autístico” OR “Autismo Infantil” OR “Síndrome de Kanner” OR “Trouble autistique”) AND (“reading” OR “leitura” OR “lectura”) AND (“phonological awareness” OR “phonological awareness skills” OR “consciência fonológica” OR “habilidades em consciência fonológica” OR “conciencia fonológica” OR “habilidades en la conciencia fonológica”))
OpenGrey	“Autism Spectrum Disorder” OR “Autistic Disorder” AND “reading” AND “phonological awareness” doctype:(U - Thesis)
Proquest	(“Autism Spectrum Disorder” OR “Autism Spectrum Disorder” OR “Autism Spectrum Disorders” OR “Autistic Disorder” OR “Autistic Disorder” OR “Kanner's Syndrome” OR “Kanner Syndrome” OR “Kanners Syndrome” OR “Infantile Autism” OR “Autism” OR “Early Infantile Autism” OR “ASD”) AND (“reading”) AND (“phonological awareness” OR “phonological awareness skills”)
PubMed	((“Autism Spectrum Disorder”[Mesh] OR “Autism Spectrum Disorder” OR “Autism Spectrum Disorders” OR “Autistic Disorder”[Mesh] OR “Autistic Disorder” OR “Kanner's Syndrome” OR “Kanner Syndrome” OR “Kanners Syndrome” OR “Infantile Autism” OR “Autism” OR “Early Infantile Autism” OR “ASD”) AND (“reading”) AND (“phonological awareness” OR “phonological awareness skills”)
Scopus	TITLE-ABS-KEY (“Autism Spectrum Disorder” OR “Autism Spectrum Disorders” OR “Autistic Disorder” OR “Kanner's Syndrome” OR “Kanner Syndrome” OR “Kanners Syndrome” OR “Infantile Autism” OR “Autism” OR “Early Infantile Autism” OR “ASD”) AND TITLE-ABS-KEY ((“reading”) AND TITLE-ABS-KEY ((“phonological awareness” OR “phonological awareness skills”)
Web of Science	(“Autism Spectrum Disorder” OR “Autism Spectrum Disorders” OR “Autistic Disorder” OR “Kanner's Syndrome” OR “Kanner Syndrome” OR “Kanners Syndrome” OR “Infantile Autism” OR “Autism” OR “Early Infantile Autism” OR “ASD”) AND (“reading”) AND (“phonological awareness” OR “phonological awareness skills”)

## Appendix B Article exclusion criteria

**Table:**

**AUTHOR (S)**	**YEAR OF PUBLICATION**	**CRITERIA**
Colcord et al.^(1)^	2019	5
Finnegan^(2)^	2019	3
Fleury^(3)^	2013	3
Uccheddu et al.^(4)^	2019	3
Whalo^(5)^	2018	3
Exclusion criteria: 1. Studies with adults, elderly or children out of school age or without a diagnosis of ASD; 2. Studies that do not report reading interventions; 3. Studies that do not assess Phonological Awareness; 4. Reviews, letters, books, conference summaries, case reports, opinion articles, technical articles and guidelines; 5. Others (type of study different from the eligibility criteria).		

References:

1. Colcord CR, Barnett JH, Zucker SH. Peer-Assisted Learning Strategy (PALS) to Address reading challenges in a second-grade student with autism spectrum disorder. DADD Online. 2019;98:1.

2. Finnegan EG. Literacy instruction for students with autism spectrum disorder in inclusive settings. DADD Online. 2019;8:72-88.

3. Fleury VP. Engaging children with autism in shared book reading: strategies for parents. Young Except Child. 2015;18(1):3-16.

4. Uccheddu S, Albertini M, Pierantoni L, Fantino S, Pirrone F. The impacts of a Reading-to-Dog Programme on attending and reading of nine children with Autism Spectrum Disorders. Animals. 2019;9(8):491.

5. Whalon K. Enhancing the reading development of learners with autism spectrum disorder. Semin Speech Lang. 2018;39(2):144-57.

## Appendix C Analysis of randomized and nonrandomized studies

**Table:**

**JBI Critical Assessment Checklist for Quasi-Experimental Studies (Non-Randomized Experimental Studies)**				
	Heimann et al.^(35)^	Kimhi et al.^(38)^	Pamparo^(42)^	Tjus et al.^(43)^
1. Is it clear in the study what is the ‘cause’ and what is the ‘effect’ (i.e. there is no confusion about which variable comes first)?	Yes	Yes	Yes	Yes
2. Were the participants included in any comparisons similar?	Yes	Yes	No	Yes
3. Were the participants included in any comparisons receiving similar treatment/care, other than the exposure or intervention of interest?	No	No	Yes	No
4. Was there a control group?	Yes	No	Yes	No
5. Were there multiple measurements of the outcome both pre and post the intervention/exposure?	Yes	Yes	Yes	Yes
6. Was follow up complete and if not, were differences between groups in terms of their follow up adequately described and analyzed?	Yes	Yes	Yes	Yes
7. Were the outcomes of participants included in any comparisons measured in the same way?	Yes	Yes	Yes	Yes
8.Were outcomes measured in a reliable way?	Yes	Yes	Yes	Yes
9. Was appropriate statistical analysis used?	Yes	Yes	Yes	Yes
Risk Rating / Percentage	Low (88.89%)	Low (77.78%)	Low (88.89%)	Low (77.78%)

(1) **Nonrandomized studies. Risk Rating:** High:49% “Yes”; Moderate: 50% to 69% “Yes”; Low: more than 70% “Yes”

**Table:**

**JBI Critical Assessment Checklist for Randomized Clinical Trials**				
	Bean et al.(32)	Gasamis(34)	Hudson et al.(37)	Nally(39)
1. Was true randomization used for assignment of participants to treatment groups?	Yes	Yes	Yes	Yes
2. Was allocation to treatment groups concealed?	No	Unclear	Yes	No
3. Were treatment groups similar at the baseline?	No	Yes	Yes	Yes
4. Were participants blind to treatment assignment?	Yes	Yes	Yes	Yes
5.Were those delivering treatment blind to treatment assignment?	Unclear	Unclear	Unclear	No
6. Were outcomes assessors blind to treatment assignment?	No	Unclear	Unclear	No
7. Were treatment groups treated identically other than the intervention of interest?	Yes	Yes	Yes	Yes
8. Was follow up complete and if not, were differences between groups in terms of their follow up adequately described and analyzed?	Yes	Yes	Yes	Yes
9. Were participants analyzed in the groups to which they were randomized?	Yes	Yes	Yes	Yes
10. Were outcomes measured in the same way for treatment groups?	Yes	Yes	Yes	Yes
11. . Were outcomes measured in a reliable way?	Yes	Yes	Yes	Yes
12.Was appropriate statistical analysis used?	Yes	Yes	Yes	Yes
13. Was the trial design appropriate, and any deviations from the standard RCT design (individual randomization, parallel groups) accounted for in the conduct and analysis of the trial?	Yes	Yes	Yes	Yes
Risk Rating / Percentage	Moderate (69.23%)	Low (76.92%)	Low (84.61%)	Low (76.92%)

(2) **Randomized Studies. Risk Rating:** High:49% “Yes”; Moderate: 50% to 69% “Yes”; Low: more than of databases, registers and other
